# Poisoning by *Atractylus gummifera* L. Roots in Grazing Cattle of a Sicilian Farm

**DOI:** 10.3390/vetsci12040305

**Published:** 2025-03-28

**Authors:** Clara Naccari, Giulia Donato, Vincenzo Naccari, Ernesto Palma, Pietro Paolo Niutta

**Affiliations:** 1 Department of Health Sciences, University “Magna Græcia” of Catanzaro, 88100 Catanzaro, Italy; palma@unicz.it; 2 Department of Veterinary Sciences, University of Messina, 98166 Messina, Italy; giudonato@unime.it (G.D.); pietro.niutta@unime.it (P.P.N.); 3 AUSL Ferrara, Dipartimento di Sanità Pubblica, Unità Operativa Complessa Sanità Animale, 44121 Ferrara, Italy; v.naccari@ausl.fe.it

**Keywords:** poisoning, *Atractylis gummifera* L., roots, cattle, grazing, Sicily, plowed pasture

## Abstract

Background: In farm animals, the main poisonings are due to the ingestion of toxic plants present in pastures, but their incidence is underestimated, even though they are responsible for serious damage to animal health and livestock production. *Atractylis gummifera* L. is a plant widespread in Mediterranean countries and in southern Italy that is responsible for serious and often fatal poisonings in both humans and animals. This investigation reports grazing cattle poisoning due to the ingestion of *Atractylis gummifera* L. roots present in a recently plowed pasture of a Sicilian farm. Methods: The investigation describes the clinical symptoms of poisoned cattle, especially the gastro-intestinal type, the alterations in liver and renal function, the course of poisoning (hyperacute–acute), and the necropsy examination of dead animals. In the surviving cattle, symptomatic drug therapy was administered, and hematological and biochemical analyses (blood, liver, and kidney profiles) were carried out during treatment. Results: The pharmacological therapy, although symptomatic, contributed to the recovery of the intoxicated cattle with a return to normal values of liver and kidney parameters and electrolyte profile 30–40 days after treatment. Conclusions: This investigation could be useful to veterinarians for the diagnosis of *Astractylus gummifera* L. root poisoning in cattle, which is also responsible for the death of some intoxicated animals, with a negative impact on livestock.

## Simple Summary

*Atractylis gummifera* L. is a perennial herbaceous plant, thorny and fragrant, with a flexible rhizome and tap root, that is present in Mediterranean countries and in the regions of southern Italy. Every year, it is responsible for serious and often fatal poisonings in humans and also in animals due to poor toxicological knowledge, the rapid evolution of intoxication, and related clinical/diagnostic difficulties. Actually, few data are present in the literature on animal poisoning due to this plant. This investigation reports the poisoning of grazing cattle by the ingestion of *Atractylis gummifera* L. roots present in a recently plowed pasture of a Sicilian farm. In particular, the symptoms (especially gastro-intestinal, hepatic and renal), the course of poisoning (hyperacute–acute), necroscopy investigations, and hematological analyses are described. This study, therefore, could be useful for farm veterinarians regarding the diagnosis of *Astractylus gummifera* L. root poisoning in cattle.

## 1. Introduction

*Atractylis gummifer* L. is a plant from the Asteraceae family that is commonly present in the flora of the Mediterranean region: particularly in North Africa (Morocco, Algeria, and Tunisia) and in southern Europe (Greece, Spain, Portugal, and France). It is also notably spread in southern Italy, including Sicily, Sardinia, and Calabria [1]. Due to its widespread distribution, it is considered a “cosmopolitan” species [2]. Known by botanical synonyms such as *Acarna gummifera* W., *Carlina gummifera* L., and *Masticogna lattiginosa* [3], the name *Atractylis* is derived from the Greek word “atractos” (spindle), referring to the stem used for making spindles, and “*gummifera*” (rubbery), due to the rubbery resin present in the plant’s root. This plant is commonly called “wild thistle” “mistletoe”, “blue thistle”, and “chameleon” because its flowers continuously change color [4,5]. Additionally, it is named using several vernacular names that vary by region. For instance, in Sicily, it is commonly known as “masticogna”, in Sardinia ‘musciurida’, in France ‘chardon a glu’ and in the Arabic region ‘el-heddah’ [1].

**Figure 1:**
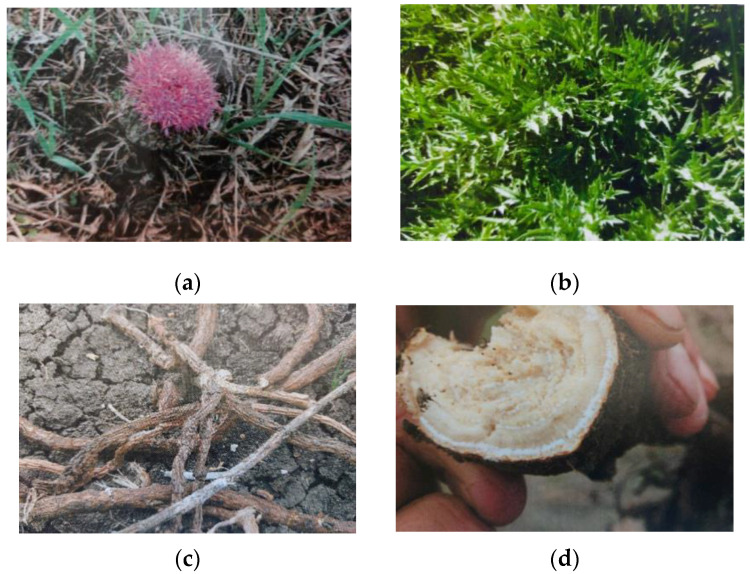
Aerial parts of *Atractylis gummifera* L.: flower (**a**), leaves (**b**), rhizomatous root, (**c**) and section of galactophore system (**d**).

*Atractylis gummifera* L. is a fragrant perennial herbaceous plant with lanceolate and spiny leaves deeply divided into spiny lobes and grouped in rosettes (30–70 mm wide) [6]. The flowers are pink, grouped in a capitulum (3–7 cm), and surrounded by bracts with three long apical spines. As the fruit ripens, a yellowish–white latex is released, which solidifies [7]. The plant has a long rhizomatous root (30–40 cm length and 7–8 cm in diameter), with numerous galactophores, that represent the plant’s milk system. These galactophores are characterized by a hard and fibrous structure and a yellowish color with long tap roots [3] (Figure 1).

It grows in arid, rocky places as well as in grassy or wooded environments and is considered a weed of meadows and pre-mountain pastures. *Atractylis gummifera* L. blooms toward the end of summer, when the plant is dry, and the rhizome is attached to a long root apparatus that sustains the plant for many years.

In folk medicine, *Atractylis gummifera* L. has been used to treat several diseases due to its various pharmacological activities, including antipyretic, diuretic, purgative, emetic, and vermifuge effects [8,9]. It has been employed in the treatment of edema, psoriasis, boils, and epilepsy as well as for stopping hemorrhages and facilitating child birth [10]. Additionally, it is used to treat ulcers, snakebite poisoning, dropsy, drowsiness, and against parasites in veterinary medicine [11]. Furthermore, its essential oil has been shown to possess important antioxidant, antifungal, and insecticidal activities [2].

Although widely employed in traditional medicine, *Atractylis gummifera* L. is also recognized as a toxic plant, as it is responsible for various poisoning cases [1,6,12]. In fact, in Mediterranean countries, cases of intoxications from *Atractylis gummifera* L. have been described since the mid-nineteenth century [6]. Poisonings in humans are accidental, because it is often collected and consumed in the place of wild artichoke and/or other edible Asteraceae. More recently, poisoning in children has been linked to the use of the root and the plant’s sweet secretions as chewing gum [13].

The toxic effects of *Atractylis gummifera* L. are attributable to its main active principles, the diterpenoid glucosides: these include atractylate of potassium, more commonly known as atractyloside (ATR) and carboxy-atractyloside (CTR), also called gummiferin, which is more toxic than atractyloside and responsible for fatal poisonings [1,6,14]. These diterpenic glucosides are present in the fresh but not dried plant, as the latter undergoes a detoxifying decarboxylation. Their content in the plant is influenced by several factors, such as climate, soil composition, genetic factors and the timing of harvest. The toxic mechanism of *Atractylis gummifera* is linked to the specific inhibitory action of atractylosides (ATR and CTR). These diterpenoid glucosides are inhibitors of mitochondrial oxidative phosphorylation, and they are able to alter the protein membrane permeabilization [15,16]. They primarily affect hepatocytes and proximal tubular epithelial cells, which contain carriers that allow ATR and ATR to cross the cell membrane. Histopathological evidence has shown marked hepatocellular and/or renal proximal tubular necrosis [17]. The plant has a liver tropism, and poisoning, whether through oral ingestion or transcutaneous absorption, can be fatal [18]. Therefore, the toxic effects of atractylosides cause hepatic and renal failure with poisoned subjects manifesting characteristic symptoms such as nausea, vomiting, epigastric and abdominal pain, diarrhea, hepatitis, anxiety, headache and convulsions, which are often followed by coma [17]. Currently, there is no specific pharmacological treatment for *A. gummifera* intoxication, and only symptomatic therapies are available.

To date, most of the available literature focuses on case reports of human poisoning, especially in children [15,18,19,20,21]. The only references concerning animals are related to a poisoning of pigs caused by the ingestion of *Atractylis gummifera* L. roots in Algeria. The poisoning was characterized by significant gastro-intestinal symptoms with salivation, vomiting, constipation, raised temperature and the death of some animals [22]. The rarity of this poisoning in livestock is due to the presence of spiny leaves in this plant and the less succulent consistency of the stem; pigs, on the other hand, are able to find and eat these roots more easily, as they frequently turn over the soil. Additionally, ingestion may occur when plowing practices bring the roots to the surface, where potentially toxic bioactive substances or contaminants accumulate [23,24] or when the animals are forced to graze for prolonged periods in the same area. Certainly, the decline of pasture quality, compounded by drought in several areas, has negatively impacted animal feed, posing a health risk to livestock [25].

The present investigation describes a case of poisoning in the grazing cattle of a Sicilian farm, which is caused by the ingestion of *Atractylis gummifera* L. roots following soil plowing.

## 2. Materials and Methods

### 2.1. Information on Poisoning of Grazing Cattle

**Figure 2:**
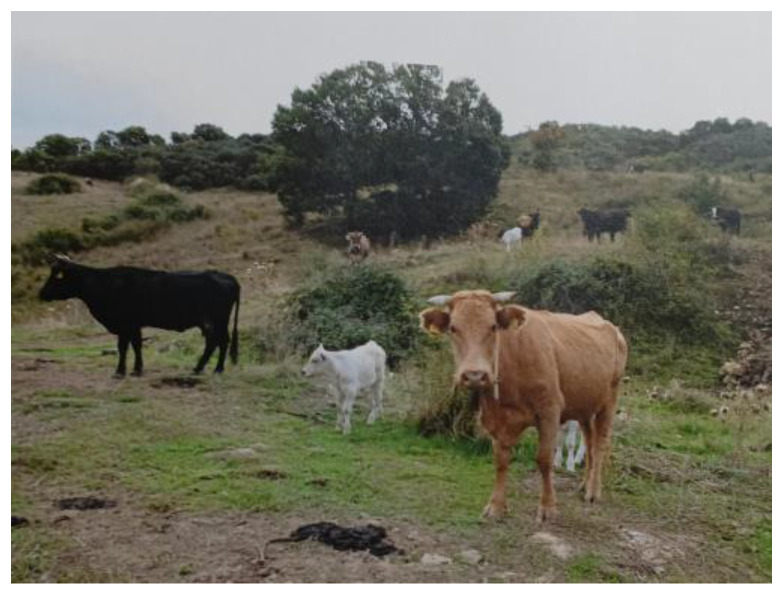
The cow–calf line farm inspected.

**Figure 3:**
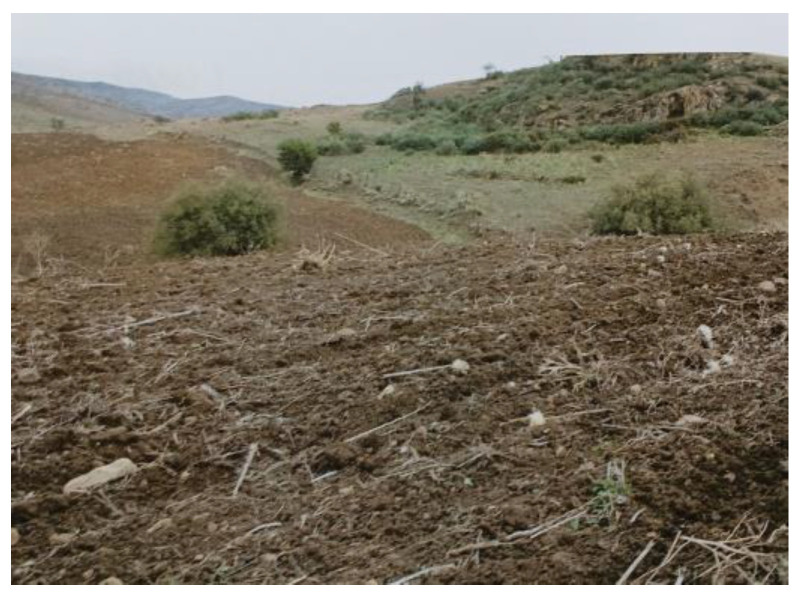
Superficial plowing of soil intended by the breeder for grazing calves.

This investigation reports a case of grazing cattle poisoning in the municipality of Randazzo, province of Catania in Sicily (Italy) due to the ingestion of *Atractylis gummifera* L. roots. The cow–calf herd that was visited by the farm veterinarian in the first half of September, consisted of 50 adult crossbred animals (Figure 2) bred in a free-range system. They were officially free from infectious diseases, according to an investigation by the National Recovery Plans of the National Health System. The animals were fed exclusively on herbaceous plants present in the pastures, constituting a polyphytic meadow typical of the territory, without any additional supplements. At the time of inspection, the pastures were particularly poor due to animal exploitation for several months. Furthermore, as part of an agricultural improvement intervention, the farmer had recently completed a very superficial plowing of the land in a plot (approximately of 1 hectare) to prepare for sowing legume crops (Figure 3). As consequence of this agricultural practice, *Atractylis gummifera* L. roots emerged from the soil. This plant, locally known in the Sicilian area as *Masticogna lattiginosa*, due to its morphological features (flowers, areal parts and roots containing rubbery resin), is widespread in the municipality where the farm is located.

### 2.2. Medical History

Regarding the medical history of the poisoned cattle, the owner reported that three days prior to the veterinarian’s inspection (requested by the owner), one cow had died and six cattle were in poor health. Of these six, one died the following day, showing the same symptoms as the previously deceased animal, while the other five showed prodromal clinical signs (Table 1). Furthermore, the owner reported that the two dead cows had been treated with Tetracycline (Panterramycin) and Imidocarb dipropionate (Carbesia), under prescription of the farm veterinarian, for a suspected tick-borne disease.

The clinical examination of the surviving cattle did not highlight hematuria, hyperthermia, or an increase in lymph nodes size but an unusual aggressive attitude, a slight hypothermia, ruminal atony and blackish diarrhea, which are symptoms that differed from those typically associated with tick-borne diseases. A thorough anamnesis revealed, as reported by the owner, that the two deceased cattle had primarily fed in the pasture on the rhizomes of *Masticogna lattiginosa* that had emerged from the soil after plowing, as observed during the inspection (Figure 3). The surviving animals had also consumed the same roots still present in the pasture.

#### Clinical Symptoms

The clinical symptoms observed in the poisoned cattle were characterized by anorexia, poor nutritional status, gastro-intestinal disorders, blackish diarrhea, a rectal temperature ranging from 36.5 to 37.5 °C, lack of rumination and the production of abundant clear urine. Abdominal auscultation revealed decreased digestive sounds in all animals, while cardiorespiratory auscultation showed an increased heart rate and signs of dyspnea. Lymph nodes were found to be normal on palpation. Furthermore, cattle showed ataxia, mydriasis, tremors, tonic spasms, aggression toward humans, hyperexcitation, sensory dullness and jaundiced mucous membranes (Table 1).

### 2.3. Necropsy Examination

A necropsy was carried out on the two cows who died from poisoning due to *Atractylis gummifera* L. roots. The liver, kidneys, lungs, intestine, spleen were examined to assess the damages associated with the intoxication from this toxic plant.

### 2.4. Clinical Analyses

Blood samples were collected from the caudal vein of the surviving cattle, using tubes containing ethylenediaminetetraacetic acid (EDTA) for the hematological profile analysis. The parameters measured included white blood cell count (WBC), red blood cell count (RBC), hemoglobin concentration (HCB), hematocrit (HCT), platelet count and leukocyte formula (neutrophils, eosinophils, basophils, lymphocytes and monocytes), using an automated hematology analyzer (HeCo Vet C; SEAC, Florence, Italy).

Additional blood samples, collected in 5 mL vacutainer tubes, were used to evaluate biochemical parameters. The following profiles were assessed: (1) liver profile—glutamate–pyruvate–transaminase (GPT or AST), glutamate oxalate transaminase (GOT or AST), gamma glutamyl transferase (GGT), alkaline phosphatase (ALP), amylase, bilirubin and glucose; (2) renal profile—urea, creatinine and total protein; (3) electrolyte content (Na, K, Mg, Ca, P and Cl) (Veterinary Automated Biochemical Analyzer BT1500VET1, FUTURLAB Limena, Italy).

### 2.5. Pharmacological Therapy

In the surviving cattle, digestive evacuation was initially performed through gastric lavage, which was followed by the administration of activated carbon to accelerate the intestinal transit. Subsequently, due to the lack of a specific pharmacological therapy for cattle poisoned by *Atractylis gummifera* L. roots, only symptomatic intravenous treatment was carried out in the upper middle third of the neck. This treatment comprised (1) sodium chloride rehydrating solution (15 mL/kg), (2) 2-methyl-phenoxy-proprionic acid (Hepagen) (30 mL for cows up to 300 kg), (3) glucose 5% solution (500 mL for cows) and (4) B vitamins complex for intramuscular injection (Table 1). Furthermore, these treated cattle, along with others from the same cow–calf farm that were asymptomatic and apparently healthy, were moved to other pastures where no agricultural practices in plowing of the soil were carried out and consequently free from *Masticogna lattiginosa* roots.

## 3. Results

### 3.1. Necropsy Examination

**Figure 4:**
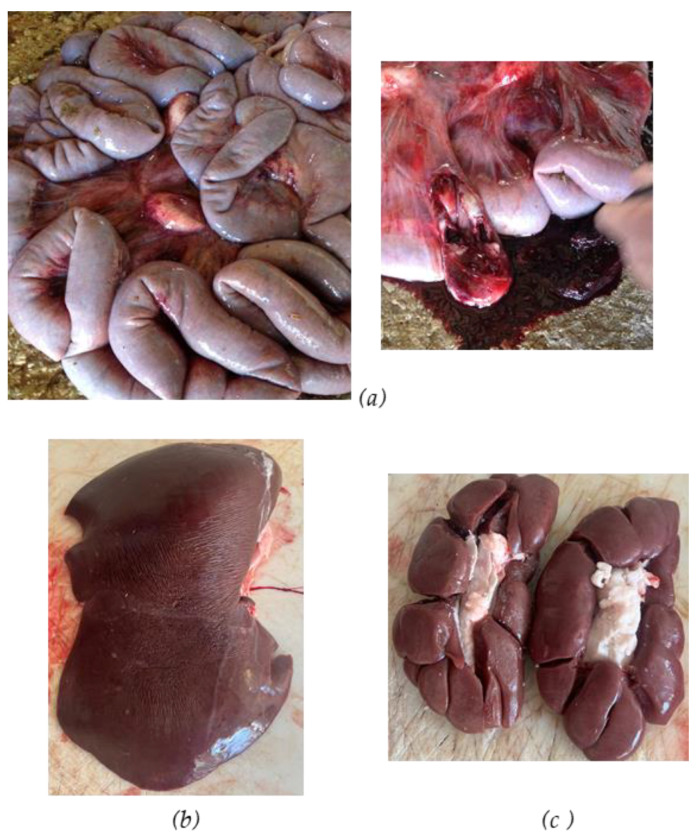
Intestine with blood and clots (**a**), congested liver and hepatomegaly (**b**) and kidneys with increased volume (**c**).

**Figure 5:**
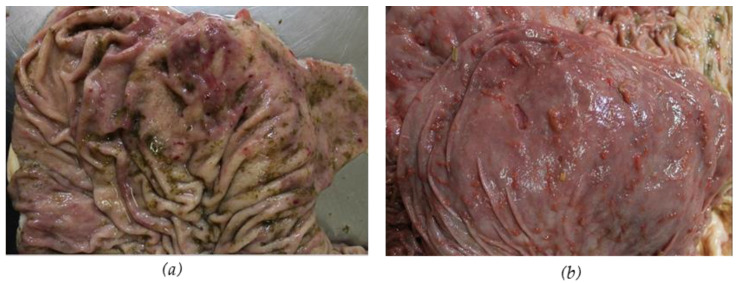
Omasum with erosions (**a**) and ulcers with crater-like appearance (**b**).

The necropsy on the two cows that died from *Atractylis gummifera* L. roots poisoning revealed the presence of peritoneal exudate, splenomegaly, liver congestion and hepatomegaly, and increased kidney volume. No visible parts of *A. gummifera* L. roots were found in the rumen content; in fact, the owner reported that the cattle had vomited undigested material containing blood the day before death. However, gastric hemorrhages were observed with the presence of wide petechiae in the rumens, reticula and particularly the omasum along with blood and clots in the intestinal tract (Figure 4 and Figure 5). In addition, the necropsy did not reveal the presence of parasites in liver and intestine.

### 3.2. Clinical–Pathological Analyses

Clinical–pathological analyses on the blood samples from the surviving cattle following *Atractylis gummifera* L. roots poisoning showed normal hematological values (Table 2). However, variations in the biochemical parameters were observed, particularly an increase in the liver (GOT, GPT, GGT and bilirubin) (Table 3) and renal (urea, creatinine and total proteins) parameters (Table 4) as well as electrolyte levels (Table 5). Subsequently, all liver and renal parameters, as well as the electrolyte levels, returned to normal values after 30–40 days of symptomatic drug treatment. The complete blood content (CBC) did not report numerical and morphological abnormalities in red and white blood cells, and also the leukocyte differential count did not show numerical alterations of corpuscular elements (neutrophils, lymphocytes, monocytes, eosinophils and basophils), thus excluding the possibility of infectious processes.

### 3.3. Pharmacological Therapy

The pharmacological therapy, although not specific but only symptomatic, contributed to the recovery of the five intoxicated cattle. This recovery was evidenced by the return to normal values of all hepatic, renal and electrolytic parameters after 30–40 days of treatment. At the same time, also, the general health of these cattle gradually improved a few days after the pharmacological treatment with complete resolution of clinical symptoms after approximately 4–5 weeks.

## 4. Discussion

Currently, the incidence of acute poisonings in animal is under-reported [26,27]. Although there are several toxic agents dangerous for animals, most of the data available in the literature are related to chronic poisonings due to the exposure to environmental pollutants in both pets and farm animals. Among companion animals, poisoning cases are mainly linked to accidental exposure to agrochemical compounds, the improper use of drugs, or intentional poisonings caused by humans [28,29].

Instead, in farm animals, the most documented poisonings are due to the ingestion of toxic substances, particularly environmental contaminants or the improper use of drugs, with a negative economic impact on livestock, apicultural, heliculture production, etc., affecting the safety and quality of animal-derived food [30,31,32]. Knowledge on the incidence of plant poisonings remains limited, although these are the most common toxic agents for both pets [33] and livestock [26,34], which are followed by mycotoxins, heavy metals, pesticides, dioxins and industrial chemicals, etc. ingested through contaminated food and water [35,36].

For grazing animals, the greatest risk of poisoning is due to the ingestion of toxic plants present in pastures [36], such as *Datura stramonium* (Jimson weed), *Senecio* spp. (ragworts and groundsels), *Quercus* spp. (oak), *Taxus baccata* (European yew), *Nerium oleander* (oleander), *Pteridium aquilinum* (brackenfern), *Robinia pseudoacacia* (black locust) and *Rhododendron* spp. (rhododendrons and azaleas) [26].

Plant poisonings in livestock are responsible for the serious direct health damage of animals, such as subclinical diseases, decreased immunity and death, or indirect damages as the result of reproduction, the reduced production of animal-derived foods and compromised food quality [30].

*Atractylis gummifera* L. is a toxic plant for both humans and animals [1,6,37] for the widespread diffusion in the Mediterranean regions; however, in the literature, cases of intoxications are reported for humans [38,39,40] and particularly in children [12,15,18,41], mainly in Marocco and Algeria. In humans, poisoning from this plant is characterized by typical alterations in biochemical parameters, such as increased levels of GOT, GPT and bilirubin, indicating hepatic damage. Clinical manifestations are related to an induced hypoglycemia, neurovegetative disorders or subsequent acute renal failure. Furthermore, mortality is associated with hepatitis due to the effect of atractyloside and carboxyatractyloside on liver tissue [4,15].

Regarding animal poisonings from *Atractylis gummifera* L. roots, few data are available in the literature. Some authors described the toxic effects of this plant on rodents, showing the lethal effectiveness of its rhizome as a valid alternative to the use of synthetic rodenticides [42,43]. The only references present in the literature on livestock species described a poisoning of pigs due to the ingestion of *Atractylis gummifera* L. roots in Algeria. The symptoms observed in pigs included serious gastro-intestinal disorders characterized by salivation, vomiting, constipation, raised temperature, and death [22,23]. In this species, the risk of poisoning from *Atractylis gummifera* L. roots is high due to their ability to frequently overturn the soil. Instead, no data are reported for other animal species, such as cows, which typically prefer more succulent herbaceous plants and without spiny leaves.

The present investigation describes a specific case report of cattle poisoning caused by the ingestion of *Atractylis gummifera* L. roots present in pastures of Sicilian region (Italy), which was identified during the clinical inspection. Particularly, the clinical history of poisoned cattle reported by the owner, the observed clinical symptoms, the necropsy findings and the CBC (hematological, hepatic, renal profile and electrolyte content) admitted to exclude possible bacterial or parasitic infections. The results documented liver and kidney alteration, which are typical of poisoning by *Astractylis gummifera* L. The clinical signs, especially gastro-intestinal and hepatic symptoms, the course of the illness (hyperacute–acute) and the prognosis in these animals were comparable to those reported in the literature for humans intoxicated by the ingestion of the same plant.

Due to the widespread diffusion of *Astractylis gummifera* L. in Mediterranean areas, particularly in southern Italy, this plant causes serious and often fatal poisonings every year, although these have been more documented in humans and underestimated in animals. The few data present in the literature, the insufficient knowledge of this toxic plant by veterinarians, and the rapid progression of poisoning make accurate clinical diagnosis challenging. Therefore, this investigation represents a useful tool for farm veterinarians to diagnose the poisoning of *Atractylis gummifera* L. roots in cattle considering key-factors: pasture livestock largely infested by *Astractylis gummifera* L. roots; the overcrowding of animals responsible of early grazing exhaustion; the animals’ ability to adapt to hostile environmental conditions, feeding also on non-succulent, spiny-leaved plants, and the lack of alternative food when forced to graze for prolonged periods in areas without adequate pastures.

Considering the severity of poisoning from *A. gummifera* L. roots in farms of Mediterranean areas, it is essential to inform owners about the importance of correct agricultural practices, such as plowing the soil in the absence of cattle and the risks of poisoning when placing animals on recently plowed land, to avoid the ingestion of the roots of this toxic plant. Additionally, it is advisable to provide supplemental feeds to cattle during the summer season when the pasture is not flourishing.

## 5. Conclusions

Most of the data present in the literature on *Atractylis gummifer* L. roots poisonings are from human cases recorded by the health services of various Mediterranean Countries. In contrast, few references are reported on the animal poisonings after the ingestion of this plant, although it is one of the most widespread toxic plants. The limited toxicological knowledge of veterinarians on toxic plants, the severity and rapid progression of this poisoning, which is often fatal in grazing animals (especially cattle) as well as always unrecognized or overlooked, and the lack of a rapid and specific diagnostic method make the diagnosis of this poisoning very difficult. Furthermore, if poisoned cattle are not promptly treated with symptomatic drugs and removed from the affected pastures, the animals inevitably die.

In conclusion, this toxicological report can be useful for farm veterinarians in diagnosing and managing cattle intoxication due to the ingestion of *Atractylis gummifera* L. roots, which is a common issue in the Mediterranean area and, particularly, to limit the severity of this intoxication responsible for the death of poisoned animals after days of agony.

To prevent this type of intoxication due to *A. gummifera* L. roots, it is advisable to inform the owners on the influence of plowing of the soil and the risks for grazing animals, especially in the Mediterranean regions where this plant is abundant, providing pasture management advice.

## Figures and Tables

**Table 1:** Generalities, clinical symptoms and symptomatic pharmacological treatment of cattle poisoned by ingestion of *Atractylis gummifera* L. roots in the pastures of a Sicilian farm.

Case Report	Generalities	Clinical Symptoms	Pharmacological Treatment
N° 1	One cross-breed cow, born on the farm, age approximately 5 years, weight 340 kg, reared in the wild, fed on pasture, rectal temperature 36.8 °C. Officially free from infectious diseases.	Anorexia, lack of rumination, tremors, jaundiced mucous membranes, aggressive behavior, hyperexcitability and dulling of the sensorium. *Died 24 h after treatment*.	2-Methyl-phenoxy-propionic Acid (Hepagen) (i.v.) Rehydrating solution sodium chloride (i.v. slow) Gluconate solution 5% (i.v.) Vitamin complex group B (Dobetin) (i.m.) * The cows in treatment, along with the others without symptoms, were moved to other pastures, where no agricultural reclamation works had been carried out.
N° 2	One cross-breed cow, born on the farm, age approximately 6 years, weight 320 kg, reared in the wild, fed on pasture, rectal temperature 37 °C. Officially free from infectious diseases.	Anorexia, poor nutritional status, tremors, tonic spasms, dulling of the sensorium, poor elasticity of the skin and subcutaneous tissue, jaundiced mucous membranes, vomit, blackish diarrhea, mydriasis and dyspnea. * *Died about 70 h after treatment*.	
N° 3	Group of n. 5 surviving cattle: Cross-breed cattle, born on the farm, age 2–3 years, weight 280–360 kg, reared in the wild, fed on pasture, rectal temperature 36.5–37.4 °C. Officially free from infectious diseases.	Clinical symptoms similar but of less intensity with respect to those described in the two deceased cows. The 5 cattle returned to normal health status (complete disappearance of clinical symptoms) approximately 30–40 days after the symptomatic treatment.	

**Table 2:** Hematological profile in calves poisoned by *Atractylis gummifera* L. roots before the pharmacological treatment.

N. Case	Hematic Parameters					Leukocyte Formula				
	RBC (×10^6^)	WBC (×10^3^)	HGB (g/dL)	HCT (%)	Platelets (×10^5^)	Neutrophils (%)	Eosinophils (%)	Basophils (%)	Lymphocytes (%)	Monocytes (%)
Range	(5–10)	(4–12)	(8–15)	(24–46)	(2–7.3)	(15–45)	(0–20)	(0–2)	(45–75)	(2–7)
1	7.6	6.5	9.5	28	2.9	36	4	2	56	2
2 *	8.5	9.2	12.4	40	4.2	39	6	1	49	5
3	8	7.8	11.7	37	45	41	3	1	52	3
4	9.5	7.3	13.5	30	6.8	34	2	0	59	5
5	9.2	9.5	12.9	36	5.4	42	8	2	45	3
6	7.8	6.4	14.2	42	6.5	28	9	1	60	2

* Died after 70 h of the pharmacological treatment.

**Table 3:** Liver profile in calves to *Atractylus gummifera* L. roots, before the pharmacological treatment, expressed according to I.S.

N. Case	Hepatic Profile					
	GPT-ALT (I.U.)	GOT-AST (I.U.)	GGT (I.U.)	ALP (I.U.)	Bilirubin (µmol/L)	Glucose (mmol/L)
Range	(17–37)	(48–100)	(18–30)	(80–156)	(1.20–1.90)	(2.1–3.9)
1	320	380	162	299	6.5	1.9
2 *	365	420	179	285	7.8	1.4
3	205	310	140	190	4.7	1.7
4	180	270	158	178	3.5	2.2
5	168	255	142	164	2.8	2.4
6	125	210	124	172	1.9	2.1

* Died after 70 h of the pharmacological treatment.

**Table 4:** Renal profile of calves poisoned by *Atractylus gummifer* L. roots, before the pharmacological treatment, expressed according to I.S.

N. Case	Renal Profile		
	Urea (mmol/L)	Creatinine (µmol/L)	Total Proteins (g/L)
Range	(3.6–9.3)	(62–97)	(59–77)
1	35.4	388	19
2 *	39.7	362	23
3	23.2	198	38
4	19.4	212	42
5	18.2	187	34
6	20.2	223	45

* Died after 70 h of the pharmacological treatment.

**Table 5:** Electrolytes content in calves poisoned by *Atractylis gummifera* L., before the pharmacological treatment, expressed according to I.S.

N. Case	Electrolytes Profile					
	Na (mmol/L)	K (mmol/L)	Mg (mmol/L)	Ca (mmol/L)	P (mmol/L)	Cl (mmol/L)
Range	(132–144)	(4–5.3)	(0.7–1.1)	(1.9–3)	(1.5–2.9)	(90–104)
1	232	7.8	0.95	1.4	1.9	95
2 *	215	6.4	1.2	3.3	2.7	87
3	135	8.2	0.84	1.8	1.85	98
4	125	4.9	0.78	2.1	1.78	101
5	145	5.5	1.05	3.2	1.48	95
6	150	4.7	0.97	2.9	2.8	102

* Died after 70 h of the pharmacological treatment.

## Data Availability

All data and results related to this study are included in the article.
